# Trajectories and characteristics of functional impairment before and after suicide attempt in young adults – a nationwide register-based cohort study

**DOI:** 10.1186/s12888-017-1567-9

**Published:** 2017-12-08

**Authors:** Mo Wang, Magnus Helgesson, Syed Rahman, Thomas Niederkrotenthaler, Ellenor Mittendorfer-Rutz

**Affiliations:** 1 Division of Insurance Medicine, Department of Clinical Neuroscience, Karolinska Institutet, SE 171 77 Stockholm, Sweden; 2 Center for Public Health, Department of Social and Preventive Medicine, Medical University Vienna, Vienna, Austria

**Keywords:** Sick leave, Disability pension, Suicide attempt, Trajectory

## Abstract

**Background:**

Despite high rates of youth suicide attempt, little is known about patterns of functional impairment in terms of sickness absence and disability pension (SA/DP) before and after an attempt. The aim was to identify SA/DP trajectories among young adults with or without suicide attempt and to describe associations of socio-demographic and clinical factors with such trajectories.

**Methods:**

This is a population-based cohort study of 5385 individuals aged 25–40 years with a first suicide attempt during 2007–2009. One control for each case without suicide attempt was matched by socio-demographic factors. Trajectories of annual SA/DP months over an eight-year period were analysed by group-based trajectory modelling. Associations between socio-demographic and clinical factors with trajectory groups were estimated by chi^2^-test and multinomial logistic regression.

**Results:**

Two groups of suicide attempters had low SA/DP levels over time (62%). One group had constantly high SA/DP levels (16%). The remaining two groups had increased SA/DP initially, which then decreased at different time points. Socio-demographic and clinical factors were associated with different trajectories (R^2^ = 0.44). Suicide attempters with low levels of SA/DP were likely to be unemployed whereas a larger proportion of those with high levels of SA/DP had psychiatric health care before the suicide attempt, particularly due to schizophrenia and non-affective psychoses or personality disorders.

**Conclusions:**

Young suicide attempters even with no/low levels of SA/DP were likely to be marginalised at the labour market. Schizophrenia/non-affective psychoses and personality disorders were important clinical factors for differentiating the levels of SA/DP among young suicide attempters.

## Background

Suicide attempt is a major and growing public health problem worldwide [1], especially among adolescents and young adults [2, 3]. Young people with suicide attempt are likely to repeat this behaviour and have an increased risk for subsequent mortality due to suicide [2]. Moreover, the long-term risk of functional impairment is increased in young suicide attempters, particularly impairment related to work incapacity, i.e. sickness absence and disability pension (SA/DP) [4]. One recent study has reported that sex, country of birth, schizophrenia, and personality disorders as well as the method of suicide attempt were associated with the subsequent risk of SA and DP in young suicide attempters [5].

Despite the evidence related to the interlinked relationship between suicide attempt and SA/DP, only a few studies have investigated the association between SA/DP and suicide attempt in the general population and in individuals with depressive disorders [6–9]. A major limitation of these studies is that they did not take the course (patterns) of SA/DP into account. When studying such associations, it needs to be considered that suicide attempters comprise a heterogeneous group in terms of aetiology, intent, socio-demographic characteristics, clinical factors such as severity of underlying mental and somatic diseases, psychosocial factors, as well as healthcare seeking behaviour [2]. Moreover, method of suicide attempt is heterogeneous, including acts such as hanging, poisoning, overdosing, or cutting [2]. SA/DP may also fluctuate over time. Trajectory analysis offers the opportunity to elucidate this heterogeneity and the differential development of time-dependent phenomena [10]. In addition, a comparison with individuals without suicide attempt can provide important insights in peculiarities of SA/DP trajectories in young suicide attempters.

### Aims

The aim of this study was to identify trajectories of SA/DP using an observation window of 8 years before and after suicide attempt in young adults and elucidate the relation of socio-demographic and clinical characteristics (i.e. underlying diagnosis-specific mental and somatic inpatient and specialised outpatient care, psychiatric medication, and method of suicide attempt) with such trajectories. Another aim was to compare the trajectories with a group of young individuals without suicide attempt.

## Methods and materials

### Study population

This study base included all individuals resident in Sweden and between 25 and 40 years of age, when treated in inpatient care due to a first suicide attempt during 2007–2009 (*N* = 6844). To assure eligibility for SA/DP particularly before suicide attempt, the age range of young suicide attempters was defined between 25 and 40 years. Individuals with previous suicide attempts from inpatient care 1987–2006 were excluded (*n* = 1396, 20.4%). Moreover, individuals with missing information on socio-demographic factors (*n* = 63, 0.9%) were excluded. The final study population consisted of 5385 individuals with first-time suicide attempt. Suicide attempt was defined based on the International Classification of Diseases (ICD) 10 codes: X60–84 and Y10-Y34. The ICD-10 codes Y10-Y34 (undetermined intent) were included in order to limit underreporting of suicide attempt and to limit temporal and geographic differences in ascertainment procedures [4, 11, 12]. A sensitivity analysis comparing estimates with and without suicide attempt due to undetermined intent yielded similar results.

Individuals between 25 and 40 years of age during 2007 and 2009 and resident in Sweden who did not have any inpatient care due to suicide attempt during 1987–2013 were used as a comparison group (*N* = 5385). For each suicide attempt case, one control was randomly selected and matched on sex, age, education, country of birth, and family situation (see categorisation in Table 1). Matching factors were measured on 31st December of the year preceding baseline (i.e. 2007–2009). The cohort entry date of the control was the date of suicide attempt of the matched case.

**Table 1:** Descriptive statistics for all women (*n* = 2878) and men (*n* = 2507) with a first suicide attempt treated in inpatient care 2007–2009 in Sweden (*N*= 5385)

*Characteristics*	All		Women		Men	
	n	%	n	%	n	%
	5385	100	2878	53.4	2507	46.6
*Socio-demographic characteristics* ^*a*^						
Age						
24–29	2329	43.2	1242	43.2	1087	43.4
30–34	1486	27.6	792	27.5	694	27.7
35–40	1570	29.2	844	29.3	726	29.0
Education (years)^*^						
Compulsory (≤9)	1730	32.1	814	28.3	916	36.5
High school (10–12)	2716	50.4	1437	49.9	1279	51.0
University (>12)	939	17.4	627	21.8	312	12.4
Country of birth^*^						
Sweden	4301	79.9	2227	77.4	2074	82.7
European countries without Sweden^b^	218	4.0	117	4.1	101	4.0
Non-European countries	866	16.1	534	18.6	332	13.2
Type of living area^c^						
Big cities	2050	38.1	1102	38.3	948	37.8
Medium sized cities	2015	37.4	1078	37.5	937	37.4
Small towns/villages	1320	24.5	698	24.3	622	24.8
Family situation^*^						
Married^d^ living without children	163	3.0	96	3.3	67	2.7
Married^d^ living with children	1158	21.5	742	25.8	416	16.6
Single^e^ living without children	3340	62.0	1368	47.5	1972	78.7
Single^e^ living with children	724	13.4	672	23.3	52	2.1
Unemployment^*^						
No	3969	73.7	2188	76.0	1781	71.0
1–180 days	1122	20.8	560	19.5	562	22.4
> 180 days	294	5.5	130	4.5	164	6.5
*Clinical characteristics*						
Somatic inpatient care^* f, g^						
No	2820	52.4	1311	45.6	1509	60.2
Yes	2565	47.6	1567	54.4	998	39.8
Specialised somatic outpatient care^* f, g^						
No	1357	25.2	641	22.3	716	28.6
Yes	4028	74.8	2237	77.7	1791	71.4
Mental diagnosis from in- or specialised outpatient care^* f, g^						
No	2369	44.0	1275	44.3	1094	43.6
Schizophrenia non-affective psychoses	152	2.8	51	1.8	93	3.7
Affective disorders	801	14.9	496	17.2	256	10.2
Substance abuse	913	17.0	258	9.0	605	24.1
Neurotic, somatoform and stress-related disorders	733	13.6	473	16.4	234	9.3
Personality disorders	213	4.0	119	4.1	49	2.0
Other mental disorders	204	3.8	103	3.6	94	3.7
Antidepressants^* h, i^						
No antidepressants	2893	53.7	1331	46.2	1562	62.3
Low doses	732	13.6	434	15.1	298	11.9
Moderate doses	1020	18.9	641	22.3	379	15.1
High doses	740	13.7	472	16.4	268	10.7
Anxiolytics^* h, i^						
No Anxiolytics	3650	67.8	1850	64.3	1800	71.8
Low doses	1288	23.9	808	28.1	480	19.1
Moderate doses	267	5.0	142	4.9	125	5.0
High doses	180	3.3	78	2.7	102	4.1
Sedatives^* h, i^						
No sedatives	3364	62.5	1695	58.9	1669	66.6
Low doses	1080	20.1	631	21.9	449	17.9
Moderate doses	555	10.3	322	11.2	233	9.3
High doses	386	7.2	230	8.0	156	6.2
Method of suicide attempt^* g, j^						
Violent suicide attempt	671	12.5	221	7.7	450	17.9
Non-violent suicide attempt	4714	87.5	2657	92.3	2057	82.1

^*^ Significant sex differences

^a^ Measured on 31-Dec of the year preceding suicide attempt

^b^ Including other Northern European countries than Sweden and the rest of EU25

^c^ Type of living area: big cities (Stockholm, Gothenburg and Malmö); medium sized cities (cities with more than 90,000 inhabitants within 30 km distance from the centre of the city); small cities/villages/rural

^d^ Married includes all living with partner; cohabiting

^e^ Single comprises divorced, separated, and widowed

^f^ Measured four years before suicide attempt

^g^ See methods section for ICD-10 codes

^h^ Measured during the year preceding the suicide attempt

^i^ See methods section for ATC codes

^j^ Measured at index suicide attempt

### Registers

Individual information was linked through registers by using the unique personal identity number. The following national registers from Swedish authorities were available for each individual retrospectively and prospectively up to 31st December 2013:

Longitudinal integration database for health insurance and labour market studies (LISA) from Statistics Sweden: age, sex, country of birth, education, family situation, type of living area, year of emigration, number of days with unemployment, sickness absence, and disability pension.
(i) date and diagnoses of in- and specialised outpatient care from the National patient register; (ii) date of death from the Cause of death register; (iii) date, defined daily dose (DDD), and Anatomical Therapeutic Chemical (ATC) Classification System code from the Drug register – all from the National Board of Health and Welfare.

### The social insurance system in Sweden

In Sweden, the Social Insurance Agency grants sickness benefits for all people from the age of 16 who have an income from work or unemployment benefits, and a reduced work capacity due to a disease or injury [13]. Employed individuals have one qualifying day without benefits and self-employed individuals may have more qualifying days according to the insurance they have chosen. Employees receive sick pay from their employers during the first 14 days. Afterwards, they receive sickness benefits from the Social Insurance Agency. After 7 days of self-certification, a physician certificate is required. DP is granted to people who have a permanently impaired work capacity due to a disease or injury. Since 2003, individuals aged 19–29 years can receive temporary DP.

### Sickness absence and disability pension

The SA and DP days were combined and the mean annual number of SA/DP days from 4 years before to 4 years after baseline was calculated. As SA and DP can be granted for part time, the number of net days was used, e.g., 2 days of half-time sickness benefits equals one net day. Then, the number of annual net days was transformed to number of annual months with SA/DP.

### Socio-demographic and clinical factors

Characteristics of individuals included socio-demographics (i.e., sex, age, education level, country of birth, type of living area, family situation, and number of days with unemployment), and clinical characteristics (i.e. diagnosis-specific mental and somatic inpatient and specialised outpatient care, psychiatric medication, and method of suicide attempt) (See Table 1).

Socio-demographics were measured on 31st December of the year preceding suicide attempt and categorised as shown in Table 1. The main diagnosis from previous in- and specialised outpatient care due to somatic disorders was measured during 4 years before suicide attempt up to the year preceding suicide attempt. The individuals with and without at least one previous somatic in- or outpatient care were coded positive and negative for this variable, respectively. The chronologically latest main mental diagnoses from in- or specialised outpatient care were categorised according to ICD-10 codes: Schizophrenia and non-affective psychoses (F20-F29); affective disorders (F30–39); substance abuse disorders (F10-F19); neurotic, stress-related and somatoform disorders (F40-F48); personality disorders (F60) and all remaining mental diagnoses (F00-F09, F50–59, F61-F69, F70-F79, F80-F89, F90-F99). Prescription of psychiatric medication, i.e. antidepressants, anxiolytics and sedatives was included and coded according to the ATC codes, namely N06A, N05B and N05C, respectively. Prescribed psychiatric medication was assessed according to DDDs during the year preceding suicide attempt and categorised into 4 groups in relation to DDDs [14]. Psychiatric medication less than 0.5 DDD was grouped as low doses; 0.5–1.5 DDD as moderate doses while high doses referred to more than 1.5 DDD [15].

Method of the index suicide attempt was defined as violent and non-violent suicide attempts [16] (Table 1). Violent suicide attempts included hanging, use of firearms or knives, jumping from height or in front of vehicles and drowning (ICD-10: X70-X82 and Y20-Y32). Non-violent attempted suicide comprised poisoning: X60-X69 and Y10-Y19 in ICD-10. There were 183 (3.4%) individuals with unspecific events/means (ICD-10: X83-X84 and Y33-Y34) who were grouped into violent suicide attempts according to the description of ICD-10 [17]. A sensitivity analysis categorising the unspecific methods into non-violent suicide attempt did not change the overall results.

### Statistical methods

Firstly, sex differences were measured in the socio-demographic and clinical characteristics among young suicide attempters by a Chi^2^ test. Then, group-based trajectory modelling was used to measure trajectories of SA/DP among individuals with or without suicide attempt from 4 years before baseline until 4 years after [18]. An annual time-scale was applied in the study, where T-4 represents 4 years before baseline and T4 equals 4 years after baseline. The method is developed in order to identify subgroups of individuals with distinct trajectories during the time of observation. Also, this method could provide the possibility to assess variation and stability over time for each identified subgroup and the proportion of individuals in each group. We tested 2–7 groups until the model of best fit was obtained, as determined by comparing the Bayesian information criterion (BIC). A model with five groups for suicide attempt cohort and a model with three groups for the comparison cohort were found as the best fitting model. The highest estimated probability was used to decide each individual’s group belonging. Côté et al. recommended an average probability of ≥0.70 for individuals of a trajectory group [19]. These probabilities for individuals in the suicide attempt cohort and the comparison cohort were 0.91 and 0.96, respectively, indicating a good fit.

Distributions of socio-demographic and clinical factors in each SA/DP trajectory group among young suicide attempters were explored by Chi^2^-test and multinomial logistic regression. The likelihood ratio test was applied to evaluate the associations between socio-demographic and clinical factors with type of trajectory group in the full model. Moreover, the strength of these associations was estimated by Nagelkerke pseudo *R* ^*2*^. Each factor was consecutively excluded and re-included from the full model in order to calculate the differences in *R* ^*2*^. These differences in *R* ^*2*^ were used as indicators of the contribution of a given factor to the full model. Additionally, the data on SA/DP for an individual who died or emigrated was considered as missing for the whole year of the event and onwards.

Data processing was performed using the statistical software SAS for Windows version 9.4 (SAS-based procedure “Traj”) [18] and SPSS for Windows version 22.0 (chi^2^-test and multinomial logistic regression).

## Results

In Table 1 descriptive statistics of the young suicide attempters with regard to characteristics on socio-demographics and clinical factors is shown for all attempters as well as stratified by sex. Of the 5385 individuals who were treated in inpatient care due to a first suicide attempt during 2007–2009, 2878 (53.4%) were women. In this group, larger proportions of the cohort were of younger age (24–29 years, 43.2%), had achieved an education at high school level (50.4%), were born in Sweden (79.9%), were single and living without children (62.0%), and were not registered as unemployed (73.7%). Men were more often single living without children than women (78.7% vs. 47.5%) while more women than men were single living with children (23.3% vs. 2.1%). Regarding clinical characteristics, the majority of suicide attempters had specialised outpatient care visits due to somatic disorders (74.8%) and used non-violent suicide attempt methods (87.5%). Substance abuse (17.0%), affective disorders (14.9%), and stress-related disorders (13.6%) were more common than other mental disorders in young adults with suicide attempt. More women than men used psychiatric medication. Additionally, more women than men had previous somatic inpatient care (54.4% vs. 39.8%) while more men had a diagnosis of substance abuse (24.1% vs. 9.0%) and used violent methods of suicide attempt (17.9% vs. 7.7%) than women (Table 1).

### Trajectory analyses

**Fig. 1:**
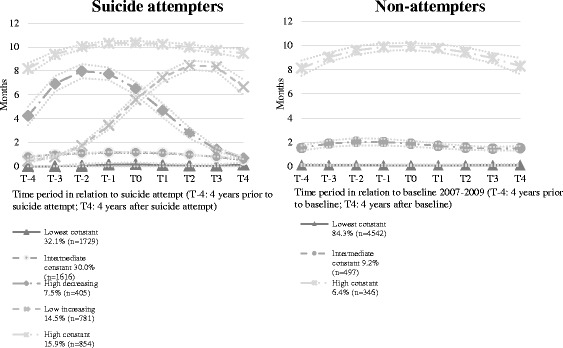
Trajectory groups of sickness absence and disability pension (SA/DP) months and percentages of individuals with suicide attempt treated from inpatient care in 2007–09 (*N* = 5385) and individuals who were living in Sweden during 2007–2009 without suicide attempt (*N* = 5385) within each trajectory group. The dotted lines represent 95% confidence intervals

Five different trajectory groups of SA/DP months for the suicide attempt cohort were identified (Fig. 1). The five groups were named as “Lowest constant”, “Intermediate constant”, “High decreasing”, “Low increasing”, and “High constant”.

Among individuals with suicide attempt, approximately one third (32.1%) belonged to the “Lowest constant” group. They constantly had no or quite few months of SA/DP over the years before and after suicide attempt. There were 30.0% of the individuals in the “Intermediate constant” group with an average of 1 month of SA/DP/year during the study period. The “High constant” group included 15.9% of the individuals. On average, they had around 10 months of SA/DP annually. The “High decreasing” group included 7.5% of the individuals. Four years before attempt, this group had approximately 4 SA/DP months per year, increasing up to 8 months at T-2, when the level of SA/DP months decreased. The “Low increasing” group included 14.5% of individuals. They had moderately low level of SA/DP months at T-4 and showed an increasing trend reaching around 8 months at T3 (Fig. 1).

The comparison cohort showed three trajectory groups over time. The three groups were named as “Lowest constant”, “Intermediate constant”, and “High constant”. The absolute majority belonged to the “Lowest constant” group (84.3%). The “Intermediate constant” group included 9.2% of individuals which is fewer than the suicide attempt group. There were only 6.4% of the individuals included in the “High constant” group (Fig. 1).

Table 2 shows distributions and associations of socio-demographic and clinical characteristics in each trajectory group among suicide attempters. All socio-demographic and clinical factors were significantly associated with the trajectory groups (*P* < 0.001) in the unadjusted analyses. After mutual adjustment in the full model, the significant results remained except for “method of suicide attempt”. Nagelkerke pseudo *R* ^*2*^ for the full model is 0.436 which indicated an association between socio-demographic and clinical factors with the trajectory groups. The differences in *R* ^*2*^ indicated that the different factors did not independently have strong effects in the full model, except for mental diagnoses before suicide attempt (Diff. in R^2^ = 0.052) and days of unemployment (Diff. in *R* ^*2*^ = 0.029), which had a more important role than other factors in the full model.

**Table 2:** Distributions and associations of socio-demographic and clinical characteristics in each trajectory group of annual months of sickness absence and disability pension (SA/DP) in individuals with suicide attempt treated in inpatient care in 2007–2009 in Sweden (*N* = 5385)

Characteristics	Lowest constant	Intermediate constant	High decreasing	Low increasing	High constant	Pearson’s Chi-Square (*p*-value)^k^	Log-likelihood test Chi-Square (*p*-value) ^l^	Diff. in R^2 *^
	n (%)	n (%)	n (%)	n (%)	n (%)			
	1729 (32.1)	1616 (30.0)	405 (7.5)	781 (14.5)	854 (15.9)			
*Socio-demographic characteristics* ^*a*^								
Sex								
Men	1014 (58.6)	708 (43.8)	148 (36.5)	294 (37.6)	343 (40.2)	161.7 (<0.001)	56.0 (<0.001)	0.007
Women	715 (41.4)	908 (56.2)	257 (63.5)	487 (62.4)	511 (59.8)			
Age								
24–29	895 (51.8)	681 (42.1)	149 (36.8)	355 (45.5)	249 (29.2)	171.4 (<0.001)	125.4 (<0.001)	0.015
30–34	447 (25.9)	483 (29.9)	115 (28.4)	207 (26.5)	234 (27.4)			
35–40	387 (22.4)	452 (28.0)	141 (34.8)	219 (28.0)	371 (43.4)			
Education (years)								
Compulsory (≤9)	667 (38.6)	389 (24.1)	132 (32.6)	208 (26.6)	334 (39.1)	154.1 (<0.001)	157.9 (<0.001)	0.019
High school (10–12)	730 (42.2)	930 (57.5)	218 (53.8)	407 (52.1)	431 (50.5)			
University (>12)	332 (19.2)	297 (18.4)	55 (13.6)	166 (21.3)	89 (10.4)			
Country of birth								
Sweden	1164 (67.3)	1377 (85.2)	354 (87.4)	655 (83.9)	751 (87.9)	259.3 (<0.001)	135.0 (<0.001)	0.016
European countries without Sweden^b^	103 (6.0)	51 (3.2)	10 (2.5)	25 (3.2)	29 (3.4)			
Non-European countries	462 (26.7)	188 (11.6)	41 (10.1)	101 (12.9)	74 (8.7)			
Type of living area^c^								
Big cities	736 (42.6)	595 (36.8)	140 (34.6)	295 (37.8)	284 (33.3)	38.6 (<0.001)	22.2 (<0.05)	0.003
Medium sized cities	636 (36.8)	593 (36.7)	161 (39.8)	303 (38.8)	322 (37.7)			
Small towns/villages	357 (20.6)	428 (26.5)	104 (25.7)	183 (23.4)	248 (29.0)			
Family situation								
Married^d^ living without children	74 (4.3)	44 (2.7)	11 (2.7)	19 (2.4)	15 (1.8)	128.4 (<0.001)	56.0 (<0.001)	0.007
Married^d^ living with children	352 (20.4)	419 (25.9)	92 (22.7)	171 (21.9)	124 (14.5)			
Single^e^ living without children	1155 (66.8)	917 (56.7)	222 (54.8)	458 (58.6)	588 (68.9)			
Single^e^ living with children	148 (8.6)	236 (14.6)	80 (19.8)	133 (17.0)	127 (14.9)			
Unemployment								
No	1177 (68.1)	1080 (66.8)	323 (79.8)	576 (73.8)	813 (95.2)	288.3 (<0.001)	248.5 (<0.001)	0.029
1–180 days	419 (24.2)	425 (26.3)	72 (17.8)	170 (21.8)	36 (4.2)			
> 180 days	133 (7.7)	111 (6.9)	10 (2.5)	35 (4.5)	<10 (0.6)			
*Clinical characteristics*								
Somatic inpatient care^f, g^								
No	1160 (67.1)	862 (53.3)	151 (37.3)	349 (44.7)	298 (34.9)	310.8 (<0.001)	99.6 (<0.001)	0.012
Yes	569 (32.9)	754 (46.7)	254 (62.7)	432 (55.3)	556 (65.1)			
Specialised somatic outpatient care^f, g^								
No	654 (37.8)	358 (22.2)	53 (13.1)	184 (23.6)	108 (12.6)	258.2 (<0.001)	49.2 (<0.001)	0.006
Yes	1075 (62.2)	1258 (77.8)	352 (86.9)	596 (76.4)	746 (87.4)			
Mental diagnosis from in- or specialised outpatient care^f, g^								
No	1049 (60.7)	826 (51.1)	106 (26.2)	235 (30.1)	153 (17.9)	1068.8 (<0.001)	435.0 (<0.001)	0.052
Schizophrenia non-affective psychoses	18 (1.0)	10 (0.6)	<10 (1.7)	32 (4.1)	85 (10.0)			
Affective disorders	122 (7.1)	245 (15.2)	88 (21.7)	198 (25.4)	148 (17.3)			
Substance abuse	340 (19.7)	255 (15.8)	85 (21.0)	107 (13.7)	126 (14.8)			
Neurotic, somatoform and stress-related disorders	143 (8.3)	219 (13.6)	81 (20.0)	130 (16.6)	160 (18.7)			
Personality disorders	15 (0.9)	28 (1.7)	26 (6.4)	47 (6.0)	97 (11.4)			
Other mental disorders	42 (2.4)	33 (2.0)	12 (3.0)	32 (4.1)	85 (10.0)			
Antidepressants^h, i^								
No antidepressants	1315 (76.1)	906 (56.1)	137 (33.8)	268 (34.3)	267 (31.3)	811.6 (<0.001)	93.6 (<0.001)	0.011
Low doses	170 (9.8)	232 (14.4)	73 (18.0)	148 (19.0)	109 (12.8)			
Moderate doses	159 (9.2)	306 (18.9)	114 (28.1)	208 (26.6)	233 (27.3)			
High doses	85 (4.9)	172 (10.6)	81 (20.0)	157 (20.1)	245 (28.7)			
Anxiolytics^h, i^								
No Anxiolytics	1443 (83.5)	1185 (73.3)	212 (52.3)	436 (55.8)	374 (43.8)	732.7 (<0.001)	47.6 (<0.001)	0.006
Low doses	241 (13.9)	373 (23.1)	128 (31.6)	273 (35.0)	273 (32.0)			
Moderate doses	28 (1.6)	36 (2.2)	41 (10.1)	50 (6.4)	112 (13.1)			
High doses	17 (1.0)	22 (1.4)	24 (5.9)	22 (2.8)	95 (11.1)			
Sedatives^h, i^								
No sedatives	1388 (80.3)	1118 (69.2)	184 (45.4)	369 (47.2)	305 (35.7)	942.1 (<0.001)	113.7 (<0.001)	0.013
Low doses	242 (14.0)	339 (21.0)	103 (25.4)	224 (28.7)	172 (20.1)			
Moderate doses	70 (4.0)	113 (7.0)	63 (15.6)	119 (15.2)	190 (22.2)			
High doses	29 (1.7)	46 (2.8)	55 (13.6)	69 (8.8)	187 (21.9)			
Method of suicide attempt^g, j^								
Violent suicide attempt	272 (15.7)	190 (11.8)	32 (7.9)	78 (10.0)	99 (11.6)	30.4 (<0.001)	5.3 (0.26)	0.001
Non-violent suicide attempt	1457 (84.3)	1426 (88.2)	373 (92.1)	703 (90.0)	755 (88.4)			

^*^ Difference in Nagelkerke pseudo R^2^ between model including tested variable and model without tested variable. Nagelkerke pseudo R^2^ for full model is 0.436

^a^ Measured on 31-Dec of the year preceding suicide attempt

^b^ Including other Northern European countries than Sweden and the rest of EU25

^c^Type of living area: big cities (Stockholm, Gothenburg and Malmö); medium sized cities (cities with more than 90,000 inhabitants within 30 km distance from the centre of the city); small cities/villages/rural

^d^ Married includes all living with partner; cohabiting

^e^ Single comprises divorced, separated, and widowed

^f^ Measured four years before suicide attempt

^g^ See methods section for ICD-10 codes

^h^ Measured during the year preceding the suicide attempt

^i^ See methods section for ATC codes

^j^ Measured at index suicide attempt

^k^ Crude model

^l^ Mutually adjusted model

Individuals in the “Lowest constant” group tended to be men, better educated, younger (24–29 years), born in other countries than Sweden, living in big cities, and unemployed while fewer individuals in this group were single living with children. The “Intermediate constant” group also had more unemployed individuals. In contrast, the “High constant” group included a larger proportion of older individuals living in small towns/villages with lower levels of education, but few individuals were unemployed. Other socio-demographic factors were relatively equally distributed among all the groups.

Regarding the clinical characteristics, more attempters in the “High constant” group had previous in- and specialised outpatient somatic care, a diagnosis of schizophrenia and non-affective psychoses or a personality disorder and use of antidepressants, anxiolytics, and sedatives, particularly high doses of use. The “High decreasing” group also included more individuals with somatic disorders, and low/moderate doses of psychiatric medication. In the “Low increasing” group, a larger proportion of individuals had affective disorders and low doses of psychiatric medication. On the contrary, the “Lowest constant” group showed less specialised somatic and mental care and less use of psychiatric medication compared to other groups (Table 2).

## Discussion

### Main findings

In this study, five different trajectories of SA/DP were identified over an 8-year period among all 5385 young individuals with a first suicide attempt treated in inpatient care during 2007–2009 in Sweden. More than two thirds of the individuals had no or few annual SA/DP months before and after suicide attempt. In contrast, 15.9% of the individuals had constantly high SA/DP months over time. The remaining two groups, including 7.5% and 14.5% in each, showed increasing SA/DP firstly and then their annual SA/DP months decreased at different time points. A comparison group of 5385 young individuals without suicide attempt showed three trajectory groups of SA/DP. The absolute majority of these individuals in group had no or very low levels of SA/DP months throughout the study period (93.5%). All socio-demographic (sex, age, education, country of birth, type of living area, family situation, and unemployment) and clinical factors (in- or specialised outpatient somatic and mental care and psychiatric medication), except for method of suicide attempt, were significantly associated with different trajectory groups (*R* ^*2*^ = 0.436, *P* < 0.05) among suicide attempters in the adjusted analyses. Young suicide attempters with low levels of SA/DP tended to be unemployed with better level of education, whereas a larger proportion of those with high levels of SA/DP had schizophrenia/non-affected psychoses or personality disorders and lower levels of education.

### Methodological considerations

To the best of our knowledge, this is the first study to investigate different trajectories of SA/DP before and after suicide attempt, compared with individuals without suicide attempt. The population-based cohort design, including all young adults aged 25–40 years in the entire country of Sweden, offered satisfactory statistical power for the analyses of trajectories of SA/DP months. Another strength was the use of high quality nationwide register data on suicide attempt, SA/DP and a wide range of socio-demographic and clinical factors [20, 21]. The use of register data also minimised the risk of recall bias regarding exposure and outcome.

Some limitations of the study are important to mention. Information on sick-leave spells <14 days among employed individuals was not available. This means that for employed individuals the number of SA days contributing to the combined number of SA/DP days might be an underestimation. Only suicide attempts that required inpatient care were included, which only covered the medically more serious cases. This means that suicide attempters who were treated at other healthcare facilities or did not seek healthcare were not included.

### Trajectories of SA/DP

Our findings showed considerable heterogeneity with regard to patterns of SA/DP before and after suicide attempt. In the suicide attempt cohort, we found that two thirds of the individuals had either no SA/DP or few months of SA/DP over time while one group had constantly high SA/DP levels (16%). The absolute majority of individuals without suicide attempt (94%) had no or very low levels of SA/DP during the 8 years of observation.

These findings are comparable to results from a recently published study, which showed five different trajectories of SA/DP before suicide [10]. However, a larger proportion of individuals (30%) in that previous study had stable high levels of SA/DP months over time before suicide, which was twice as much as among suicide attempters in the current study. Also, this group of individuals had around 11 months of SA/DP during 4 years before suicide [10], which was moderately higher than the corresponding group in our findings. On the other hand, there was a larger proportion of young suicide attempters with constantly no or low level of SA/DP months (62%) in our study compared to the low SA/DP trajectory (46%) before suicide [10]. In the current study, only young adults aged between 25 and 40 at the time of suicide attempt were included. As a result of the peculiarities of the social insurance system in Sweden, younger individuals might be less eligible to SA due to a lack of labour market attachment [13, 22]. That could explain why high SA/DP was less frequent in this study compared to the study on suicide completers. Other explanations might be differences in the morbidity level between suicide attempters and suicide completers [2, 23, 24].

Our results further indicate that SA/DP trajectories can be characterised by different socio-demographic and clinical factors. The specific mental diagnosis before suicide attempt showed the strongest associations in the full model. Mental disorders are very common diagnoses behind SA/DP [13]. Moreover, mental disorders represent the most important risk factors for suicidal behaviour [2, 23]. Therefore, it is reasonable to find that a larger proportion of individuals in the “High constant” group had psychiatric health care before their suicide attempt, particularly due to schizophrenia, non-affective psychoses or personality disorders and high doses of antidepressants, anxiolytics, and sedatives. The “High decreasing” group with relatively high levels of SA/DP months before suicide attempt also had low/moderate doses of psychiatric medication. This might reflect a higher morbidity level and lower levels of work capacity requiring more SA/DP days and increasing the risk of suicide attempt. In addition, the majority of individuals in these groups had previous specialised somatic healthcare. This is in line with studies on somatic comorbidity in suicide attempters [2].

On the other hand, the “Lowest constant” group consisted of individuals with fewer previous somatic and mental healthcare and less use of psychiatric medication in comparison with other groups. It might be due to the fact that the group also include more younger individuals (below 30 years of age) and more men. Young people may have better health conditions than older ones and thus use less healthcare and medication, however, young men might also be less likely to seek help and disclose health problems compared to their female counterparts [25, 26]. Therefore, undetected mental or somatic disorders are likely in this group. Furthermore, a larger proportion of individuals in this group was born in a non-European country. It might be possible that they were less eligible to SA due to their lower level of labour market attachment or had less access to healthcare than individuals from the host country [27]. On the other hand, a larger proportion of unemployed individuals were found in the “Lowest constant” and the “Intermediate constant” group compared to other groups. This might indicate that young suicide attempters were marginalised at labour market even though the levels of SA/DP were low. This result is in line with previous research which has demonstrated an association between unemployment and suicide attempt on the one hand and with SA/DP on the other hand [2, 28].

Interestingly, two fluctuating trajectory groups, “High decreasing” and “Low increasing” were found in this study. The “Low increasing” group included more individuals with a diagnosis of affective disorders and low doses of psychiatric medication use, which might be associated with subsequent increasing SA/DP months and suicide attempt. On the other hand, it is surprising that about 8% of individuals had increasing SA/DP months in the beginning of the observation period and their SA/DP months declined from 2 years before suicide attempt. A considerable proportion of this group of individuals had previous in- or specialised outpatient somatic health care, substance abuse, and prescribed psychiatric medication. The decreasing SA/DP months might reflect improvement in health conditions, decreased help-seeking or inadequate treatment or rehabilitation. Suicide attempt as an impulsive behaviour could be also associated with other factors than healthcare characteristics [2]. Thus, further studies are required to gain more knowledge in this trajectory group.

## Conclusions

This study showed five different trajectories of SA/DP over a period of 8 years before and after suicide attempt among young adults. While two thirds of the suicide attempters had no or low levels of SA/DP over time, 15% had constantly high levels. In contrast, the absolute majority (94%) of young adults without suicide attempt showed no or low levels of SA/DP during the observation time. Socio-demographic and clinical factors were associated with different SA/DP trajectories, especially earlier diagnosed schizophrenia/non-affective psychoses, personality disorders, and unemployment. It seems that marginalisation at the labour market is considerable for suicide attempters even though the levels of SA/DP are low.

## Abbreviations

ATC: Anatomical therapeutic chemical
BIC: Bayesian information criterion
DDD: Defined daily dose
DP: Disability pension
ICD: International classification of diseases
LISA: Longitudinal integration database for health insurance and labour market studies
SA: Sickness absence
